# Cellular Microenvironment Influences the Ability of Mammary Epithelia to Undergo Cell Cycle

**DOI:** 10.1371/journal.pone.0018144

**Published:** 2011-03-29

**Authors:** Alexa I. Jeanes, Apolinar Maya-Mendoza, Charles H. Streuli

**Affiliations:** Wellcome Trust Centre for Cell-Matrix Research, Faculty of Life Sciences, University of Manchester, Manchester, United Kingdom; University of Sao Paulo – USP, Brazil

## Abstract

The use of cell culture models is a principal and fundamental technology used in understanding how mammalian cells work. However, for some cell types such as mammary epithelia, the lines selected for extended culture are often transformed or have chromosomal abnormalities, while primary cultures have such a curtailed lifespan that their use is restricted. For example, mammary luminal epithelial cells (MECs) are used to study mechanisms of breast cancer, but the proliferation of primary cell cultures is highly limited. Here we describe the establishment of a new culture system to allow extended analysis of cultures of primary mouse MECs. In 2D monolayer culture, primary MECs showed a burst of proliferation 2–3 days post isolation, after which cell cycle decreased substantially. Addition of mammary epithelial growth factors, such as Epidermal Growth Factor, Fibroblast Growth Factor-2, Hepatocyte Growth Factor, and Receptor Activator for Nuclear Factor κB Ligand, or extracellular matrix proteins did not maintain their proliferation potential, neither did replating the cells to increase the mitogenic response. However, culturing MECs directly after tissue extraction in a 3D microenvironment consisting of basement membrane proteins, extended the time in culture in which the cells could proliferate. Our data reveal that the cellular microenvironment has profound effects on the proliferative properties of the mammary epithelia and is dominant over growth factors. Moreover, manipulating the cellular environment using this novel method can maintain the proliferative potential of primary MECs, thus enabling cell cycle to be studied as an endpoint after gene transfer or gene deletion experiments.

## Introduction

Understanding the mechanisms of cell cycle regulation in normal breast epithelia is essential for deciphering the defects of breast cancer, and therefore for developing new therapies to treat the disease. We have discovered, using molecular genetic approaches, that the β1-integrin gene is necessary for the proliferation of normal luminal epithelial cells within the breast [1], [2]. Gene deletion studies have also shown that β1-integrin is required for breast cancer progression [3], [4]. Thus the factors controlling cell cycle regulation in breast epithelia are broader than locally acting and systemic growth factors and hormones. Luminal epithelial cells are the precursors of most breast cancers and it is therefore important to determine the mechanisms linking integrins with proliferative responses in this cell type. However, this poses logistical issues because of the problems associated with growing luminal cells in tissue culture.

Mammary epithelial cells (MECs) are widely used to study epithelial cells in general, as well as mammary specific functions such lactation. Although much work has been done using immortalised cell lines, primary luminal MECs isolated directly from the mouse or human mammary gland are a preferred model because their phenotype is more similar to cells *in vivo* [5], [6], without the numerous changes associated with immortalisation that can affect cell behaviour [7], [8]. Indeed, studying mechanisms of mammary development and function, such as ductal morphogenesis and alveolar differentiation, are now possible with the use of 3D culture techniques using reconstituted basement membrane such as 3D BM-matrix [9], [10].

Unfortunately, normal primary mammary epithelial cells (MECs) have a poor growth response to conventional 2D culture conditions, proliferating slowly, and undergoing apoptosis [11] or becoming senescent [12]. While human MECs can be propagated for a limited number of times, mouse MECs behave differently and do not proliferate well after the first passage. Occasionally cells can emerge from senescence through immortalisation, where changes in genomic structure including telomere rescue occur [13]. However, immortalisation disrupts the normal cell cycle regulatory mechanisms, such as phosphorylation of Rb protein, limiting the appropriateness of using immortalised lines for studying cell cycle mechanisms. Moreover, MEC lines established from mice often form hyperplasias when injected into mammary fat pads [14]. Thus it is pressing to uncover ways of extending the experimentally useful proliferation window in normal primary MEC cell cultures.

In this paper, we have explored growth factor and extracellular matrix (ECM) requirements for maximising the time frame of luminal MEC proliferation in culture. For most of the experiments herein, we have used luminal cells isolated from pregnant mouse mammary glands, firstly because this is the time in development when maximal proliferation *in vivo* occurs, and secondly because cancers largely arise within the alveolar lobules rather than within ducts themselves [15]. We find that manipulating the cellular microenvironment alters the ability of such cells to undergo cell cycle. This provides both new understanding of cell cycle regulation in breast, and a practical solution for determining gene function in this process.

## Results

### S-phase cell cycle progression in primary mouse mammary epithelial cells in conventional 2D culture

**Figure 1:**
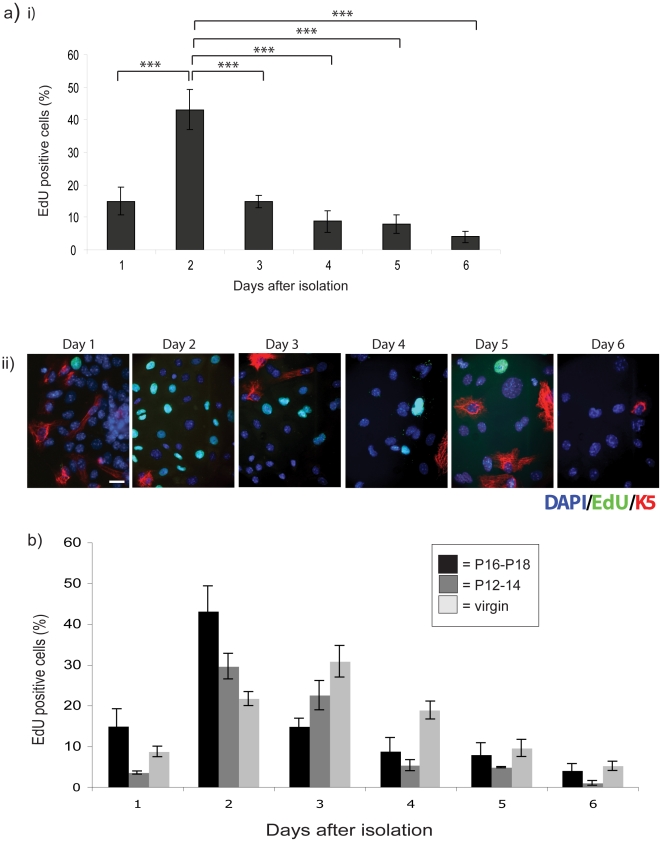
Primary MECs display limited proliferation potential in 2D culture. (a) MECs were isolated from pregnant mice and plated onto collagen I coated coverslips in complete media. (i) The percentage of proliferating cells was determined by EdU incorporation on each day for 6-d after isolation. Statistical significance determined by ANOVA is indicated: *** = p<0.001. (ii) Cells were co-stained with keratin 5 to detect the myoepithelial cells. Scale bar: 13 µm. (b) MECs from pregnancy days 12–14 (mid-grey) and 10 week old virgin (light-grey) mice were isolated and treated as above, and their proliferation compared to the cells isolated at pregnancy days 16–18 (black) by EdU incorporation. There were no significant differences in proliferation between MECs from ducts and alveoli within each time point (not shown on the graph).

Cell cycle studies have traditionally been conducted on conventional 2D substrata. Therefore we initially examined the proportion of MECs in S-phase that were isolated from pregnant mouse mammary gland (P16–P18) and cultured on collagen I-coated dishes (Fig. 1a). Throughout these studies, we used primary cultures of normal non-immortalised MECs, studied directly after isolation or after one passage [16]; we assessed S-phase cell cycle progression in proliferating cells by 5-ethynyl-2′-deoxyuridine (EdU) incorporation into DNA, followed by detection with fluorescent azide [17].

Approximately 40% of the cells were in S-phase 2–3 days following isolation, but this fell to less than 10% cycling cells for the remaining time of analysis. Both luminal and myoepithelial cells are isolated during the preparation of MECs (which are largely free of fibroblasts and endothelial cells). The proportion of myoepithelial cells were quantified by counting the number of cells positive for the myoepithelial cell marker, keratin 5, and was found to be no more than 5–7% [16].

Similar cell cycle characteristics were also seen in MECs isolated at different stages of pregnancy and development (Fig. 1b). The profile was not significantly different in MECs isolated at pregnancy days P12–14 with those from P16–18, and cells from virgin mammary glands showed maximal cell cycle progression at day 3 of culture, thereafter decreasing and remaining low from 5 days of culture (light grey bars).

Although the differences in proliferation between MECs from ducts and alveoli were non-significant at the different time points, in each case levels of proliferation ≥20% were only seen for the first 2–4 d of culture, after which the percentage of cells in S-phase fell to ≤10% for as long as the cells survived in standard planar culture (Fig. 1b). We previously showed that after 4 d in culture, luminal MECs begin to undergo substantial apoptosis [11].

### Growth factors or different ECM proteins cannot extend or enhance the percentage of proliferating primary MECs in 2D culture

Mechanisms of cell cycle are usually studied in cell lines in which cells have undergone phenotypic, and sometimes genetic, changes to promote extended lifespan. We wished to determine methods that could reveal how the cell cycle is regulated in non-immortalised, very early MEC cultures, and therefore examined ways of extending this brief window of proliferation that characterised the first 3–5 days of primary cell culture, using cells from pregnant mammary glands.

**Figure 2:**
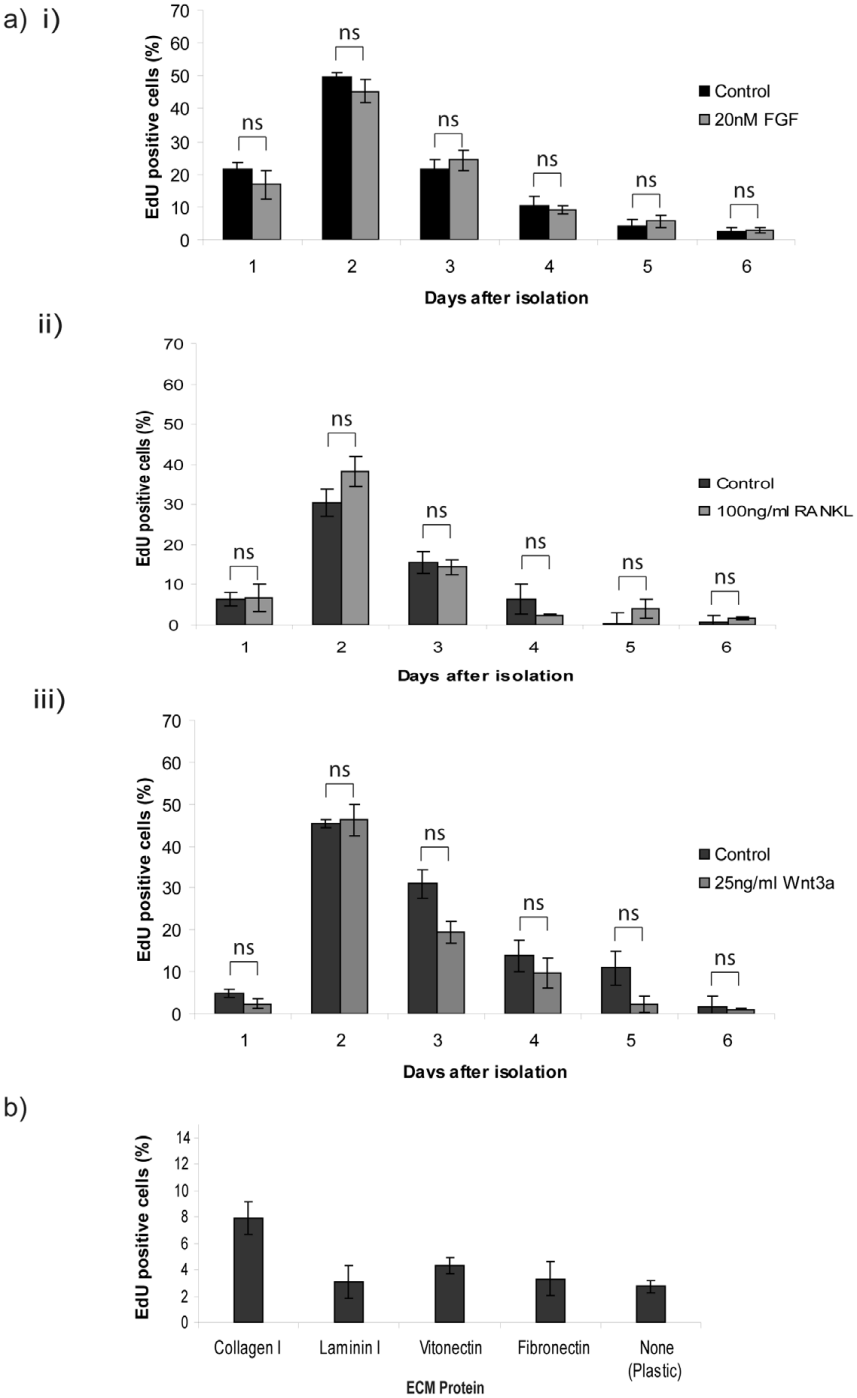
Proliferation is not enhanced or extended by mammary gland growth factors or different ECM proteins. (a) MECs were treated with (i) FGF, (ii) RANKL, and (iii) Wnt3a at the time of plating for the duration of culturing, and the proliferation was determined each day over 6-d. In each case, statistical analysis in control and growth factor treated MECs was compared by ANOVA. The pairs of samples were found to be not significantly different (ns). At each time point, the difference in %EdU-positive cells was compared to that at day-2 (i.e. the maximum), and found to be significant, p<0.001 (not shown on the graphs). (b) MECs were plated on dishes coated with collagen I, laminin, vitronectin, fibronectin, or on plastic, and proliferation was determined 4-d after plating. The difference in %EdU-positive cells between collagen and the other substrata was: plastic p<0.001; vitronectin p<0.01; laminin and fibronectin p<0.1 (not shown on graph).

A key cell cycle determinant of breast epithelia is growth factors. Despite previous studies indicating that EGF and insulin are sufficient for the growth of normal MECs [18], these factors together with serum were not able to maintain more than 15% cells in S-phase after day-4 of culture (Fig 1a). Moreover, adding fresh growth factors did not reactivate cell cycle. In the mammary gland *in vivo*, the growth factors that stimulate proliferation during puberty and pregnancy include IGFs (whose action is mimicked by high levels of insulin in our cultures), Amphiregulin (whose effects are mediated by EGF in culture), Fibroblast Growth Factor-2 (basic FGF) [19], Receptor Activator for Nuclear Factor κ B Ligand (RANKL) [20], [21] and Wnt [22]. To determine if these factors promote cell cycle in MECs, cells were cultured using amounts of bFGF, RANKL, or Wnt3a known to have a physiological effect (Fig. 2a). None of the growth factors showed any significant effect on the percentage of cells in S-phase compared to control cells.

In addition to growth factors, ECM proteins can alter the proliferative response of luminal MECs [23]. MECs were cultured on different ECM proteins and proliferation was assessed 4 d after plating cells on collagen I, laminin I, vitronectin, fibronectin or directly on the plastic of the culture dish (Fig. 2b). The proportion of proliferating cells on collagen I was approximately 8% (i.e. as in Fig. 1a and Fig. 2ai), but less than 5% on the other substrata.

Thus, the proliferation potential of MECs cannot be extended or enhanced by manipulating the culture medium by addition of growth factors, or by altering the 2D ECM protein substratum.

### Replating does not restore the proliferation potential of primary MECs in 2D culture

**Figure 3:**
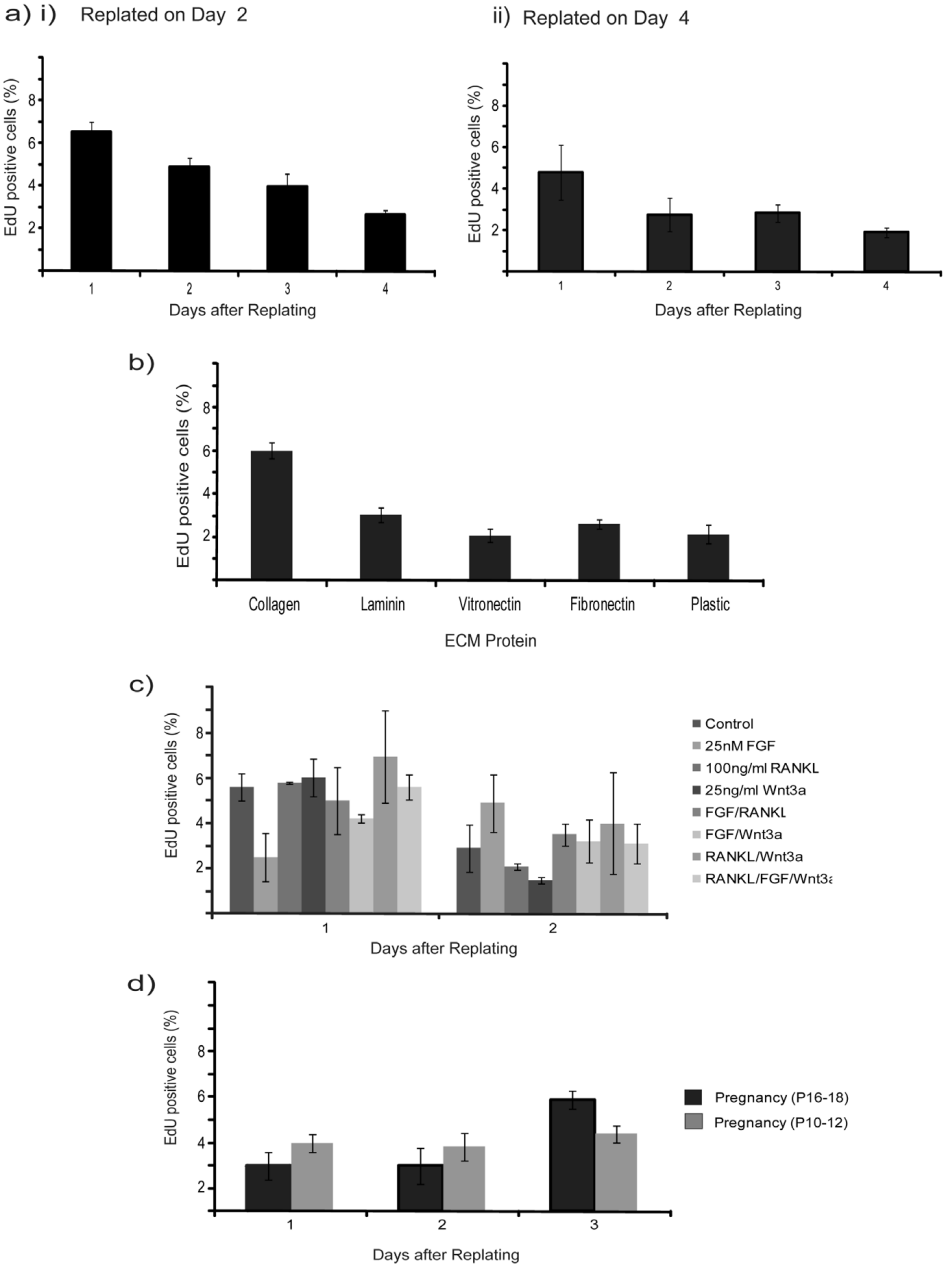
MEC proliferation is largely ablated by replating. (a) MECs were cultured on collagen I for (i) 2-d or (ii) 4-d before trypsinising and replating onto fresh collagen I coated plates, and proliferation was then assessed daily for 4-d. Note that in each of these graphs in Fig. 3, the % of proliferating cells was very low, i.e. <6%, and we did not note significant differences between the values (not shown on graph). (b) Cells replated 2-d after isolation were plated onto different ECM proteins and proliferation determined 24-h after replating. (c) Replated cells were treated with FGF, RANKL or Wnt3a in various combinations, and proliferation was determined 1-d or 2-d after replating. (d) Proliferation of replated cells originally isolated from either day 10–12 or day 16–18 pregnant mice was compared.

Contact inhibition and spatial restriction are negative regulators of epithelial cell cycle. During cell-cell contact, the ligation of E-cadherin up-regulates the cell cycle inhibitor p27, blocking proliferation [24]. Since MECs on collagen I were nearly confluent at day 4 when the proliferation levels were very low (Fig. 1a) we reasoned that releasing contact inhibition by replating the cells might reactivate cell cycle. MECs were replated at a density of approximately 2.5×10^4^ cells per cm^2^, either when the peak of cells were in S-phase, i.e. ∼45% at 2-days after isolation, or once proliferation had decreased, i.e. <10%, after 4 days. Proliferation was analysed each day for 4 days following replating, but at no point were more than 6% of cells in S-phase (Fig. 3a).

Replating onto different ECM also did not promote proliferation (Fig. 3b); similarly the addition of HGF [25] (not shown), bFGF, RANKL, or Wnt3a to complete media either alone or in combination, to replated cells failed to promote proliferation (Fig. 3c). MECs harvested from different pregnancy time points also failed to proliferate following replating (Fig. 3d).

These results show that monolayer cultured MECs do not cease proliferating because they become confluent, but rather they enter an apparently irreversible quiescence. In contrast to cell lines, this quiescence cannot be rescued by trypsinising and replating the cells and appears to be irreversible under 2D growth conditions. Moreover, the cells do not undergo senescence, as judged by β-gal staining (data not shown).

### Altering the cellular microenvironment prolongs the proliferation potential of primary MECs

**Figure 4:**
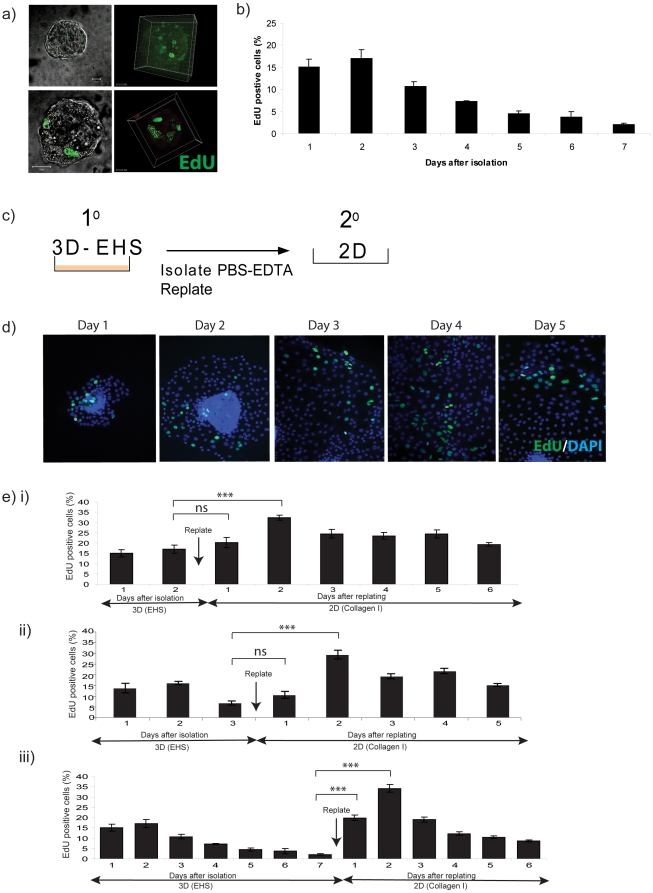
3D culture maintains the potential of MECs to proliferate when they are subsequently returned to conventional 2D culture. (a) MECs were plated directly onto 3D BM-matrix, treated with EdU each day, and confocal projection images were obtained. (b) The percentage of EdU positive nuclei was determined in comparison to the total number of DAPI-staining cells, each day over 6-d. (c) 3D acini were isolated after 2-d culture in 3D BM-matrix by washing in PBS-EDTA to dissolve the matrix followed by centrifugation to recover the acini, which were replated onto 2D collagen I. (d) The cells proliferated and emigrated from the acini. Proliferation in the 2D cultures was determined by EdU incorporation each day after replating. (e) Proliferation was determined each day in 3D culture and then in the resulting 2D cultures. Cells were cultured in 3D for (i) 2-d, (ii) 3-d, or (iii) 7-d before the acini were isolated and replated onto 2D collagen I. Statistical significance determined by ANOVA is indicated: *** = p<0.001. Here we have only included the statistical differences in the %EdU positive cells between the last day of culture in 3D and the first 2 days of culture on 2D.

3D ECMs such as a BM-matrix have been used extensively to the study cell behaviour because they bestow an environment more similar to that found *in vivo* than planar dishes [26], [27], [28]. Consequently, we explored whether 3D culture might provide an opportunity to maintain or extend the proliferation potential of primary cultures. P16–18 MECs form spherical acini when they are cultured in 3D BM-matrix (Fig. 4a). The proliferation rate of primary cells from late pregnancy in these 3D structures over the course of 7 days had a similar profile to that cultured in 2D, with an initial burst of cells in S-phase at day 2, which steadily decreased (Fig. 4b). Notably, the behaviour of primary MECs in 3D culture is different to non-malignant human MEC lines such as MCF-10A, which proliferate steadily over a period of 7–10 days before exiting cell cycle [29].

The culture of primary MECs in 3D BM-matrix mimics some of the conditions the cells are exposed to *in vivo*, with the presence of basement membrane proteins and a 3D structure. We hypothesised that, despite this loss in proliferation whilst culturing in 3D, the intrinsic potential to undergo cell cycle may not be lost in those conditions. We therefore tested whether the proliferation potential of primary MECs could be maintained in 3D culture over a period of several days, such that when acini were isolated and replated onto 2D ECM, the cells efficiently enter S-phase again.

Mammary acini were isolated from the 3D BM-matrix in sterile PBS containing 5 mM EDTA, which retained the acinar structure of the MECs but removed the BM-matrix, and then transferred to pre-coated collagen I culture dishes (Fig. 4c). The cells migrated out of the 3D structure, proliferated and formed a monolayer on the 2D collagen (Fig. 4d).

When MECs were cultured as 3D acini for 2 days and then replated into 2D, the number of cells in S-phase peaked 2 days later, similar to primary MECs (Fig. 4ei). Interestingly, these cells maintained a high level of cell cycle (i.e. more than 20%) for a longer time period than cells plated onto a 2D substratum directly after tissue isolation (compare Fig. 4ei with Fig 1a). The duration of 3D culture before replating the cells did not affect the ability to MECs to proliferate when entering 2D culture (Fig. 4eii, iii). For example, even after culture in 3D for 7-days when proliferation was reduced to 2%, the cells showed a significant and dramatic cell cycle burst when replated into 2D cultures (Fig. 4eiii). Indeed, regardless of the time that the primary MECs were cultured in 3D, the cells showed an increase in proliferation after transfer to 2D conditions, such that ∼30% of the cells were in cycle 2 days after replating. Interestingly, for cells that had been in 3D culture for longer, the 2D proliferation kinetics and amplitude returned to the normal 2D profile (compare Fig. 4eiii with Fig. 1a).

Removing MECs from their *in vivo* environment to standard 2D culture conditions disables their ability to proliferate beyond a few days. However mimicking *in vivo* conditions using 3D BM-matrix, maintained the proliferative potential of the MECs for at least 7 days, so that after replating into 2D culture, a significantly higher proportion of cells were able to enter cell cycle again.

### 3D culture maintains, but does not reset the ability for MECs to proliferate

Cells in 2D culture lost the ability to proliferate after 3–4 days, and replating the cells in 2D could not restore this. In contrast, cells initially cultured in 3D retained the ability to proliferate when they were replated in 2D. We therefore determined whether replating 2D cultures into 3D BM-matrix could reset the ability of MECs to proliferate at the levels seen immediately after isolating cells from tissue, or whether it purely maintained the proliferation potential of the cells at the point that they were put into 3D BM-matrix.

**Figure 5:**
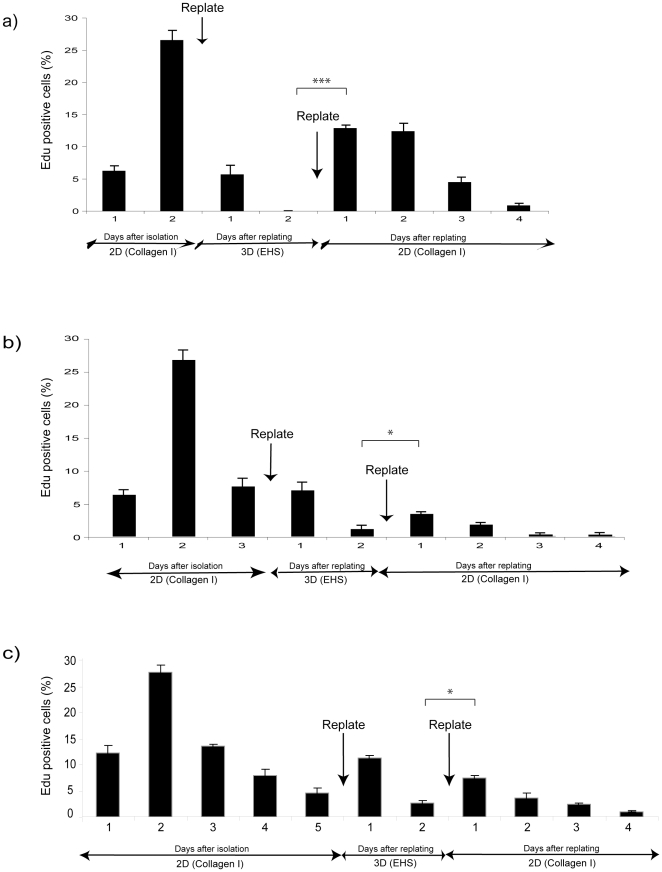
3D culture retains, but does not reset, the ability of cells to proliferate subsequently in 2D. MECs cultured in 2D for (a) 2-d, (b) 3-d, or (c) 5-d were replated onto 3D BM-matrix for 2-d, the acini were then isolated using PBS-EDTA and subsequently replated back onto 2D collagen-coated dishes. Proliferation was assessed each day. Statistical differences (ANOVA) in the %EdU positive cells between the last day of culture in 3D and the first day of culture on 2D are indicated.

Primary MECs were cultured for a varying amount of time in 2D on collagen, then replated in 3D BM-Matrix. After 48 h, the MECs were isolated from 3D BM-matrix using PBS/EDTA and replated back onto 2D collagen for a further 4 days. The proliferation was analysed by EdU over the course of the experiment (Fig. 5).

When the cells were transferred to 3D BM-matrix at the peak of their proliferation in 2D (i.e. day 2), and then re-cultured in 2D, the cells were able to attain a proliferation rate of 13%. This was maintained for 2 days and declined thereafter (Fig. 5a). In contrast, when the cells had lost their ability to progress through S-phase after 3–5 days in 2D, then transferred to 3D BM-matrix for 2 days before being replated back onto 2D collagen, the proliferation level was much lower with less than 10% of the cells being EdU positive (Fig. 5b and c).

These results show that 3D BM-matrix maintains the cells' proliferation potential at the point when they were placed into 3D BM-matrix. For cells that had lost their ability to proliferate in 2D culture, it did not reset the cells to the state they were in when they were isolated from the mammary gland. Thus, the cellular microenvironment has a dramatic effect on determining the intrinsic ability of the MECs to permanently exit cell cycle.

### Plating cells in 3D culture allows for effective gene deletion using the CreER system

One strategy to understand proliferative mechanisms is to delete the genes or deplete expression of genes encoding cell cycle regulators. While plasmid transfection is a standard methodology in established cell lines, this is not possible in primary MECs, where <0.5% cells can be transfected by any means that we have tried (unpublished data). Primary cell cultures with limited lifespans require more sophisticated techniques, such as the use of Cre-mediated gene deletion of floxed-alleles. However, because proteins frequently require several days to be turned over following ablation of the genes that encode them, the window of opportunity for doing this in MECs while maintaining proliferative potential is extremely limited. The ability of MECs to retain their proliferative potential in 3D culture over 7 days, by manipulating their environment, provides an opportunity to delete genes and their encoded proteins before replating the cells in 2D culture in order to analyse the resulting phenotype.

**Figure 6:**
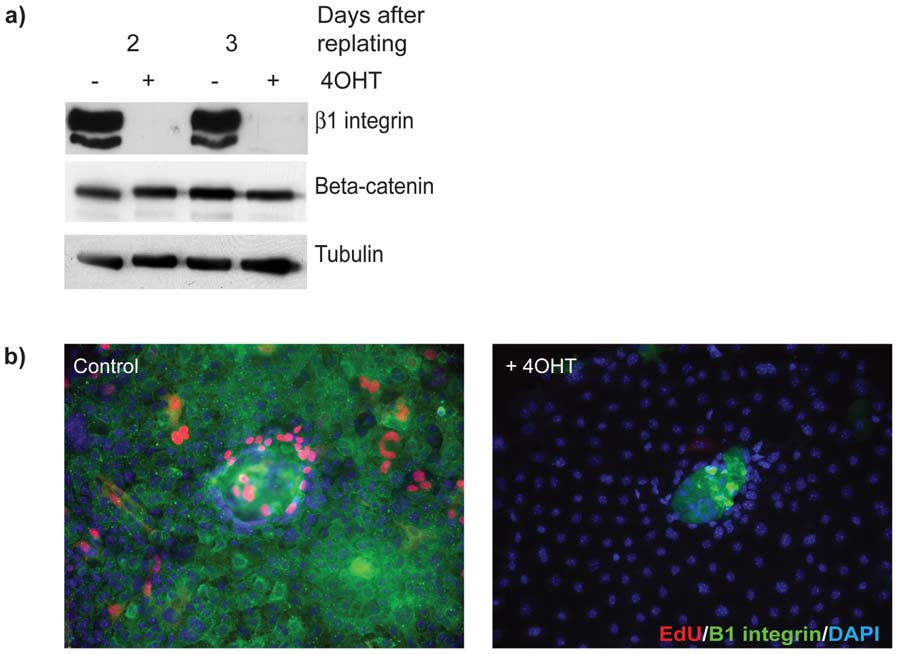
Effective Cre-mediated β1-integrin deletion in primary MECs. (a) MECs from β1^fx/fx^;CreER™ mice were cultured on 3D BM-matrix for 3-d in the presence or absence of 4OHT, isolated using PBS-EDTA and replated onto collagen I coated plates for 2-d or 3-d, where they formed a cell monolayer. These cells have lost β1-integrin expression, shown by immunoblotting. (b) The integrin-deleted cells showed defective proliferation by EdU incorporation.

As proof of principle that this approach works, we tested if MECs in which the β1-integrin gene had been excised in 3D culture, showed integrin protein loss and cell cycle defects after replating the cells onto 2D substrata (i.e. using the culture sequence similar to that shown in Fig. 4ii). MECs from Itgβ1^fx/fx^;CreER™ mice were cultured in 3D BM-Matrix in the presence or absence of 4-hydroxytamixofen (4OHT) for 3 days, then replated onto 2D ECM in normal medium. 2 days later, the 4OHT-treated cells showed complete β1-integrin removal and a corresponding reduction in cell cycle, while the control cells proliferated strongly (Fig 6). This methodology now provides a robust strategy for examining the mechanisms behind integrin-mediated control of cell cycle, which we have followed up [30].

## Discussion

We have discovered that manipulating ECM dimensionality can alter the lifespan of primary luminal MECs in culture. These cells almost completely lose their ability to undergo cell cycle in conventional monolayer culture after 3 days, and this can be overcome by replating the cells in 3D, but not by growth factors. Thus, it is possible to increase the life span of the MECs by culturing them in a 3D matrix before plating in a 2D surface. This indicates that there is a dominance of cellular microenvironment over growth factors for controlling epithelial cell cycle. However, 3D culture is not able to reset proliferative potential when cells have already lost this capacity after 2D culture. The extended window of proliferation afforded by 3D culture, prior to plating cells in monolayer, provides an operational advantage for genetic manipulation of primary cultures of non-immortalised MECs, because it permits sufficient time for gene deletion, e.g. using *Cre*-mediated recombination or mRNA depletion with shRNA, to enable mechanistic studies on cell cycle regulation.

### Limitations in primary MEC proliferation in 2D culture

Various strategies to culture luminal MECs from both mouse and human have been used in order to study mechanisms of growth regulation and cancer progression in breast. However, the normal culture environment has a profoundly negative effect on the ability of MECs to proliferate [23], [31]. Luminal MECs have a limited lifespan *in vitro*, and previous studies noted that mouse MEC proliferation in the first passage reduced to less than 10% [32].

We have examined the proliferation profile of primary luminal MECs in 2D monolayer culture over a 6 day period. The most effective ECM substratum was collagen I, and soluble factors included serum, EGF, insulin and hydrocortisone [23], [33]. The cells showed a burst of proliferation during the first 2–3 days of culture, which subsequently dropped to an almost undetectable level. The low level of proliferation could not be rescued by addition of growth factors that are now known to have a key role in mammary gland development *in vivo*, such as RANKL, FGF-2, and Wnt3a, or by replating the cells to release the contact inhibition. Indeed, we did not identify any conventional procedures that could be used to promote luminal MEC growth in primary culture or after passage, which is a similar finding to that of previous investigators.

### Strategies to increase MEC proliferation

Overcoming senescence and identifying conditions that promote continuous cellular proliferation are basic requirements for cells to grow *ex vivo*. Mouse luminal MEC lines have been established that retain the ability to differentiate and form ducts after *in vivo* transplantation, but this is extremely rare, and cell passage usually results in cessation of growth or acquisition of tumorigenic characteristics [34]. In some cases, culturing cells in collagen gels for several weeks before plating in 2D has been successful, but the majority of these lines are genetically altered because they form hyperplasias *in vivo* [14]. The generation of MEC lines can be assisted by cellular immortalisation techniques such as expression of SV40 large T antigen or TERT [35], [36], or by providing conditions in which rare variants with increased proliferation potential can emerge [13]. However, immortalisation often disrupts cell cycle regulatory mechanisms, or results in epigenetic or genomic changes that allow cells to escape quiescence [7]. It is therefore not an ideal state from which to study the proliferation mechanisms of normal cells [8].

There is an intimate relationship between luminal MECs and other cell types within the mammary gland. For example, stromal-epithelial interactions regulate epithelial growth, survival, migration and differentiation [37], [38]. Co-culturing primary MECs with other cell types can recreate the normal organisation of breast lobules [39], [40]. Moreover, lethally irradiated cells of the immortal LA7 rat mammary tumour line [19], fibroblasts [41] or the mammary fat pad [42] have been used to increase the growth of MECs. This however is not experimentally practicable when mechanisms of MEC behaviour are studied in isolation, or when suitable markers are not available to distinguish between the cell types allowing the unambiguous identification of the epithelial cells within the culture model [43]. Stromal cells do not contact luminal cells directly *in vivo* because they are separated from them by basement membrane, and in some cases the effect they have on epithelial cell behaviour has been attributed to the production of ECM proteins [44]. We therefore tested whether providing MECs with basement membrane proteins in the form of 3D-matrix might modulate the proliferative potential of MECs.

### Manipulating the 3D culture environment changes MEC proliferative characteristics

The culture microenvironment has a vast effect on cellular morphology and function. For example, it is well established that alveolar luminal MECs grown in monolayer cannot differentiate, whereas the equivalent cells cultured in 3D BM-matrix express tissue-specific genes [9]. This is because of a complex requirement for integrin-laminin interactions to license the prolactin/Stat5 pathway [45].

We have now shown that culturing alveolar luminal MECs for up to 7 days in 3D BM-matrix allows the proliferation of bulk cell cultures to be studied in subsequent 2D culture over a longer timeframe than when cells are initially plated in monolayer. Interestingly, the kinetics of proliferation in 3D culture are similar to those in 2D culture, with MECs showing an initial increase of cell cycle 2–3 days after plating, followed by a decline. Thus 3D culture *per se* does not alter proliferation response. However, the cells have a remarkable plasticity in 3D culture. They can be maintained for at least one week under those conditions and when they are replated into 2D culture they show a substantial burst of proliferation, similar to the cells isolated from tissue. In contrast, culture for up to a week in 2D culture results in permanent cessation of growth. This is not quiescence because the cells cannot be stimulated to enter the cell cycle again, and the cells do not express senescence markers. Rather, they eventually become apoptotic because they do not have the correct ECM survival signals [11], [46].

Although the virgin luminal MECs have similar growth kinetics to alveolar cells in 2D culture, interestingly these cells continue to proliferate slowly over several weeks in 3D culture to form ducts (Cheung and Streuli, unpublished). It may be that our results using 3D cultures in this paper reflect the use of alveolar cells, which have a limited proliferation potential *in vivo*. The expansion of the alveolar cells in pregnancy is dramatic, however it ceases once the gland has become filled with cells at around the start of lactation, and the natural subsequent response is for the cells to undergo apoptosis during weaning.

Together, our results show that 2D culture conditions are not suitable for extended growth of primary mouse MECs, whether they are isolated from virgin or pregnant animals. In contrast, 3D culture provides a microenvironment in which the cells maintain their proliferative potential. Alveolar cells exit cell cycle as they form acini, but if they are removed from this environment, they can proliferate again for a window of time, reflecting the plasticity of MECs.

### A strategy to study cell cycle mechanisms in primary cell culture

The limited proliferation potential of primary MECs causes significant technical problems for dissecting the molecular basis of cell cycle control in these cells. New strategies for elucidating gene function include the use of Cre-Lox gene deletion and silencing with shRNAs. However, both of these techniques rely on a sufficient time being available for the endogenous gene products to be turned over by the targeted cell. In some cases, deleting or depleting long-lived gene products involved in cell cycle regulation may not be compatible with the 2–3 days available for maximum S-phase potential in primary MECs. For example, cell adhesion plays an important role in regulating proliferation, yet many cell adhesion proteins have long half-lives.

Our new method for extending the proliferation window of MECs now provides opportunities for dissecting how the cell cycle is controlled in normal non-immortalised epithelia [30]. For example, it affords sufficient time for genes to be deleted using the Cre-LoxP system, as illustrated in Fig 6. In that case, a floxed gene was deleted by 4OHT-activation of Cre recombinase, thereby enabling the consequences of gene deletion to be studied after replating the cells. The example presented pertains to the beta1-inetgrin gene, but the method would be suitable for primary MECs from any mouse harbouring flox alleles in combination with transgenic CreER™.

In addition, this method of replating cells to maintain cells for prolonged periods is also valuable for other types of genetic modification. An increasingly used technique for primary cell cultures is the use of lentiviral-mediated gene transfer. We have now established this methodology for gene silencing with shRNAmiRs and for gene overexpression using lentivirus constructs. For example, by exploiting the replating time schedule shown in Fig 5a, we have found that we can achieve high efficiency lentiviral gene transfer by infecting cells in 2D, then transferring the cells to 3D culture conditions for direct analysis, or for subsequent replating in order to study the consequences of gene modification in 2D cultures (Wu and Streuli, unpublished).

Both of these methods now provide tractable means of genetic analysis in primary MEC culture, which up to now have been hampered by extremely low efficiencies of transfection and retroviral gene transfer.

## Materials and Methods

### Animals

Mice were housed and maintained according to the University of Manchester and UK Home Office guidelines for animal research. Animals were bred under Home Office Project Licence 40/3155, and approved by the University of Manchester ethical review process. Experiments were conducted according to S1 killing of the Animals Scientific Procedures Act 1986.

### Primary mouse mammary epithelial cell culture

MECs were isolated and cultured from ICR mice as described [11]. All studies in this paper used primary MEC cultures. In some studies, we used cells from the Itgβ1^fx/fx^;CreER™ mouse line, which was derived by crossing the Itgβ1^fx/fx^ and CreER™ mouse lines [47], [48]. All cells were from pregnant mice (pregnancy days P16–18), unless otherwise stated.

The cultures dishes were prepared as follows; Rat tail collagen I was diluted in cold PBS to give a final concentration of 10 µg/ml and dishes were coated with 100 µl per cm^2^ dish area, resulting in a coating density of 10 µg/cm^2^. The extracellular matrix proteins laminin (12 µg/ml) fibronectin (12 µg/ml) and vitronectin (3 µg/ml) were purchased from Sigma UK. The proteins were diluted to the specified concentrations in cold PBS. The ECM protein/PBS mixture was incubated overnight at 4°C or 1–2 hours at 37°C. The dishes were washed three times with cold PBS. Engelbreth-Holm-Swarm mouse sarcoma basement membrane matrix (3D BM-matrix) was purchased from BD Biosciences. 3D BM-matrix was defrosted on ice and spread over the culture plates using the end of a blue tip before incubating at 37°C for 30 min to set. Both 2D ECM proteins and 3D BM-matrix coated plates were conditioned with the serum/fetuin mix, containing double the concentration of growth factors and antibiotics, for approximately 3–4 hours at 37°C before plating the cells.

Cells were cultured in complete growth media containing 5 µg/ml insulin, 1 µg/ml hydrocortisone (Sigma), 3 ng/ml epidermal growth factor (EGF),10% foetal calf serum (Biowest), 50 U/ml penicillin/streptomycin, 0.25 µg/ml fungizone and 50 µg/ml gentamycin in Ham's F12 medium (Gibco). All cultures were maintained at 37°C in a 5% CO2 atmosphere. Cre-mediated deletion of β1-integrin was achieved by treating Itgβ1^fx/fx^;CreER™ MECs with 100 nM 4OHT.

### Isolation of mammary gland acini from 3D BM-matrix using PBS-EDTA

MECs cultured on 3D BM-matrix were incubated in sterile PBS/5 mM EDTA, scraped off the dish using a cell scraper or end of a blue tip, transferred to Falcon tubes, and incubated on ice for 5 min with gentle shaking [49]. This, together with wash from dishes, was transferred to a fresh Falcon tube and centrifuged (42×g, 3 min). Resulting acini were resuspended in fresh PBS-EDTA, incubated on ice for 5 min, recentrifuged, washed in fresh media, and resuspended in the final volume of complete media and plated onto collagen I-coated dishes. Most acini adhered to the substrata within a few hours, and the cells migrated as sheets of cells onto the dishes.

### Immunofluorescence staining in 2D and 3D

MECs were fixed in 4% paraformaldehyde/PBS (10 min, RT), and permeabilised in 0.2% Triton X100/PBS (5 min, RT). The blocking reagent was 5% goat serum (Biosera), Primary antibodies that recognise cytokeratin 5 (AF138) (Covance), and alpha-tubulin (T-9026) (Sigma), and secondary anti-rabbit Alexa 594 and anti-mouse Alexa 488 antibodies (Invitrogen). Nuclei were stained with Hoechst 33342 (1∶10000 in PBS) for 2–3 min. The coverslips were mounted onto twin frosted glass slides using ProLong® Gold antifade reagent (Invitrogen). The cells were visualised using a Zeiss Axioplan2 microscope equipped with a Hamamatsu ORCA-ER digital camera and images were captured and processed using OpenLab software (Improvision UK).

### 2D and 3D EdU proliferation assay

MECs were pulsed with 10 µM EdU, added to current culture media, for 8 hours to measure DNA synthesis [50]. The cells were fixed in 4% paraformaldehyde, permeabilised using 0.5% Triton X100, and blocked 10% goat serum in PBS. EdU was detected by incubating cells with fresh Click-iT™ buffer for approximately 30 min, protected from light (Click-iT™ EdU Alexa Fluor® 488 Imaging Kit # C10083 from Invitrogen). The cells were washed once using the wash solution provided and a normal immunofluorescence protocol was used to co-stain for other proteins, protecting from light at all times. The cells were counted blind and the number of EdU labelled nuclei calculated as a percentage of the total DAPI stained nuclei. An average of 2000 cells was counted per experiment for a minimum of 3 independent experiments.
